# Knowledge and Practice of Primary Health Care Providers in the Management of Asymptomatic Hyperuricemia and Gout in the Qassim Region of Saudi Arabia

**DOI:** 10.7759/cureus.30976

**Published:** 2022-11-01

**Authors:** Elias A Alraqibah, Feras M Alharbi, Nawaf S Alharbi, Mohammad I Aldekhail, Khaled M Alahmadi, Mohammed A Alresheedi, Mohammad I AlKhattaf

**Affiliations:** 1 Rheumatology, Qassim University, Buraydah, SAU; 2 Internal Medicine, Qassim University, Buraydah, SAU; 3 General Practice, Qassim University, Buraydah, SAU; 4 Internal Medicine: Pediatrics, Qassim University, Buraydah, SAU

**Keywords:** knowledge, phps, primary healthcare providers, asymptomatic hyperuricemia, gout

## Abstract

Introduction & Aim

The most prevalent type of inflammatory arthritis is gout. It develops because of hyperuricemia, which makes monosodium urate (MSU) crystals accumulate in the joints. However, hyperuricemia does not always cause gout.

Methodology

The following is a cross-sectional study conducted in the Qassim region of Saudi Arabia. 133 PHPs in this region were given a self-administered questionnaire through an online survey. The questionnaire included four sections: Demographic data (i.e., age, gender, years of experience) Knowledge of asymptomatic hyperuricemia; Management practices of asymptomatic hyperuricemia; Knowledge and practice of gout management

Results

One hundred thirty-three primary healthcare providers took part (males 63.9%; females 36.1%). The proportion of PHPs who attended continuing medical education (CME) on AH or gout was 32.3%. Moreover, 67.7% already knew the guidelines for managing AH or gout. PHPs’ level of knowledge regarding the management of AH and gout was good (45.9%), but their level of practice was poor (23.3%). Greater experience and CME attendance on AH and gout contributed to better understanding and higher practice scores.

Conclusion

Although PHPs’ knowledge of managing AH and gout was adequate, this did not reflect in their practice. Physicians with more years of experience who attended CME on AH and gout demonstrated better knowledge and practice than the rest of the PHPs. It is necessary to address the gaps in the practice of our PHPs, which could be done through in-depth training about AH and gout. Our study could guide other researchers to assess the gaps in other clinical practices that PHPs face.

## Introduction

The most prevalent type of inflammatory arthritis is gout, which is more common in men than women. Gout prevalence varies worldwide depending on the demographic analyzed and the research methods utilized, ranging from <1% to 6.8%. Thus, PHPs must know how to diagnose and manage it [1], Dehlin et al., 2020. Gouty arthritis results from monosodium urate (MSU) crystal deposition in the joints due to high plasma uric acid levels by Bardin & Richette, 2014 [2]. A serum urate level greater than 6.8 mg/dL is considered hyperuricemia Martillo et al., 2013 [3].

Asymptomatic hyperuricemia (AH) is a form of hyperuricemia where symptoms of gout do not occur. It constitutes 90% of patients who have hyperuricemia. Therefore, only some hyperuricemia patients are gout patients. However, AH is considered the first stage of advanced gout, which may last more than ten years before the flares develop [2], Bardin & Richette, 2014. Although AH is not a disease, it can predict the risk of cardiovascular disease and experiencing flares in the next five years [4], Mandell, 2008. The uric acid level depends on the balance between endogenous purine production and excretion via the kidneys. In 90% of patients, underexcretion on behalf of the kidneys caused their gout. On the other hand, 10% of the patients developed gout due to over-production by Ragab et al., 2017) [5].

Studies involving 838 primary care physicians have shown that a small percentage (11.8%) of participants stated that they were aware of gout therapy recommendations. Regarding the management of acute gout, when there is an acute flare, 84% of physicians stated that they would not aspirate the affected joint, and 86.6% would order a serum urate level test. 58.8% suggested colchicine for acute gout management, and 50.5% would use non-steroidal anti-inflammatory drugs (NSAIDs). However, 45% suggested using glucocorticoids for acute gout. Most physicians (84.6%) would not initiate urate-lowering therapy (ULT) during an acute attack [6], Harrold et al., 2013. (73.5%) of participants advised their patients to modify their diet. Another cross-sectional study, conducted in January 2021 at the University of Split School of Medicine, Croatia, involved 336 primary care physicians. A significant percentage of practitioners (42.3%) needed to familiarize themselves with European recommendations for gout management. However, around half of the participants stated they were satisfied with their approach to gout patients, and 67.2% of physicians based their practice on their clinical experience. Around half of them remarked that they had not read a single scientific paper about gout in the past year. However, physicians who had read at least one paper on gout management in the past year showed better results in the knowledge questions; furthermore, around half correctly identified drugs that could lower or elevate serum uric acid levels Furlan et al. in 2021 [7].

In a local aspect, a cross-sectional study of PHPs in Jeddah, from December 2017 to May 2018, has highlighted a less-than-adequate knowledge of AH and gout. Only 32.8% of involved physicians possessed a good understanding of AH. Regarding AH management, around half of the physicians misprescribed ULT by recommending either a long-term or short-term use of such treatment. Nevertheless, the majority (88.1%) asked the patients to make lifestyle changes and have a low-purine diet. The most frequent barriers that have been self-reported for good practice were a lack of awareness and knowledge about guidelines in the management of gout and AH [8], Alqarni & Hassan, 2018. 

In addition, another cross-sectional study was conducted on PHPs in Bisha in 2021. There were 142 physicians (86.6%) possessing adequate knowledge of AH. Moreover, most physicians reported awareness of guidelines for managing AH and gout (82.3%). For good practice only (43.9%) had good practice regarding asymptomatic hyperuricemia. Additionally, years of experience and good knowledge contributed positively towards managing AH by Alharthi, 2021 [9].

This study will evaluate the knowledge and practice among primary healthcare physicians in the Qassim region. It will specifically assess the management of gout and asymptomatic hyperuricemia. This will include the restriction of physicians to the current guidelines, considering patient history to diagnose gout, and deciding when to start ULT. These objectives have yet to be investigated in the Qassim region.

## Materials and methods

Methodology

Study Design

This study was held in the Qassim region. This study followed a cross-sectional survey-based method. The study population includes primary healthcare physicians, from those specializing in family medicine to general practitioners. PHPs outside of the Qassim region, rheumatologists, and internal medicine physicians (including residents and specialists) were excluded from our research. The PHPs in the Qassim region were all eligible to participate in the study and the physicians’ responses to the questionnaire constituted our study population.

Data Collection Plan

The questionnaire for the study was a modified self-designed electronic questionnaire. It was adapted from similar studies performed elsewhere and formulated according to the recent guidelines to address our study objective. The data will be managed and analyzed by the 24th version of IBM SPSS statistics software.

Ethical Considerations

The informed consent document indicated the purpose of the study and the rights of the participant for confidentiality. It also explained that they could withdraw at any moment with no commitment to the study team and to contact the study team if they had any queries. By giving each participant a code number just for analysis, we were able to ensure their privacy. Participants received no incentives or benefits. Approval from the institutional review board (IRB) was obtained prior to beginning this study.

This study obtained ethical approval from Qassim Regional Research Ethics Committee (Number 1443-880505)

Scoring

Five questions determined the overall knowledge of PHPs regarding managing AH and gout. The correct answers were coded with “1” and the incorrect answers with “0”. Question 5 was a multiple response answer with three correct answers, giving a total knowledge of 7 items. The total knowledge score was obtained by adding all 7 items and a total score range from 0 to 7 has been generated which generally means that the higher the score the higher the knowledge toward the management of asymptomatic hyperuricemia and gout. A cutoff point of the total score determined the overall level of knowledge; a score of less than 50% showed poor knowledge, between 50%-75% demonstrated moderate knowledge, and more than 75% was a marker of a good level of knowledge.

Determining the practice of PHPs toward managing AH and gout was obtained from 8-item questionnaires, where the correct answers were coded with “1” and the incorrect answers with “0”. Item 2 (three correct answers) and item 3 (three correct answers) gave a total practice item of 12. The total practice score was acquired by adding all 12 items, generating a score range from 0 to 12. Therefore, the greater the score, the greater the practice of managing AH and gout. A cutoff point of the total score determined the level of practice; a score of less than 50% showed a poor level of practice, between 50%-75% demonstrated moderate practice, and more than 75% was a marker of good practice.

Statistical Analysis

Mean ± standard deviation were used to present quantitative variables and quantitative variables were displayed using percentages and numbers. The knowledge and practice scores were compared to the socio-demographic characteristics by using the Mann-Whitney U test as well as the Kruskal-Wallis test. Normality tests were conducted using the Shapiro-Wilk test and the Kolmogorov-Smirnov test. Both knowledge and practice scores follow an abnormal distribution. Thus, non-parametric tests were applied. The Pearson correlation coefficient was used to determine the correlation between the knowledge and practice scores. P-value <0.05 was considered statistically significant. The Statistical Packages for Software Sciences (SPSS) version 26 was used for all statistical analyses, Armonk, New York, IBM Corporation.

## Results

This cross-sectional survey recruited 133 PHPs. Table 1 describes the socio-demographic characteristics of the PHPs. The most common age group ranged from 35 to 44 (45.1%), and PHPs with more than ten years of experience constituted 45.1%. The proportion of PHPs who were aware of the guidelines on managing AH and gout was 67.7%.

**Table 1:** Socio-demographic characteristics of primary healthcare providers (PHPs) (n=133)

Study Variables	N (%)
Age group	
25–34 years	35 (26.3%)
35–44 years	60 (45.1%)
45–54 years	29 (21.8%)
55–64 years	09 (06.8%)
Gender	
Male	85 (63.9%)
Female	48 (36.1%)
Medical specialty	
General practitioner	75 (56.4%)
Family medicine	58 (43.6%)
Years of experience	
<5 years	26 (19.5%)
5–10 years	47 (35.3%)
>10 years	60 (45.1%)
Attended continuing medical education (CME) on asymptomatic hyperuricemia (AH) or gout	
Yes	43 (32.3%)
No	90 (67.7%)
Read about asymptomatic hyperuricemia (AH) or gout in the last year	
Yes	94 (70.7%)
No	39 (29.3%)
Aware of guidelines on the management of asymptomatic hyperuricemia (AH) or gout	
Yes	90 (67.7%)
No	43 (32.3%)

The assessment of PHPs’ knowledge in managing AH and gout is presented in Table 2. It was revealed that the overall mean knowledge score was 5.18 (SD 1.39), with poor, moderate, and good knowledge levels detected among 13.5%, 40.6%, and 45.9% of the participants, respectively.

**Table 2:** Assessment of primary care providers’ (PHPs) knowledge in the management of asymptomatic hyperuricemia and gout (n=133) † Variable with multiple response answers. * Indicates correct answer.

Statement	N (%)	
Which of the following values is correct regarding asymptomatic hyperuricemia (AH)?	
Serum uric acid level is >7 mg/dl in males and >6 in females *	94 (70.7%)
Serum uric acid level is >6 mg/dl in both males and females	18 (13.5%)
Serum uric acid level is >6 mg/dl in males and >7 in females	21 (15.8%)
Uric acid precipitation causes an inflammatory response	
Correct *	115 (86.5%)
Incorrect	09 (06.8%)
I do not know	09 (06.8%)
Asymptomatic hyperuricemia (AH) always progresses to gouty arthritis	
Correct	37 (27.8%)
Incorrect *	78 (58.6%)
I do not know	18 (13.5%)
Asymptomatic hyperuricemia (AH) always needs treatment	
Correct	27 (20.3%)
Incorrect *	98 (73.7%)
I do not know	08 (06.0%)
Which of the following is correct regarding the pathogenesis of asymptomatic hyperuricemia (AH)? ^†^	
Increase production of urate *	103 (77.4%)
Decrease in renal excretion of urate *	99 (74.4%)
Dietary source *	102 (76.7%)
Increase renal excretion of urate	13 (09.8%)
Total knowledge score (mean ± SD)	5.18 ± 1.39
Level of knowledge	
Poor	18 (13.5%)
Moderate	54 (40.6%)
Good	61 (45.9%)

Regarding PHPs’ practice in managing AH and gout, the poor practice was observed regarding the preferred treatment to perform during the acute setting of the first gouty attack, as only 22.6% indicated joint aspiration. Nearly two-thirds (65.4%) of the PHPs were correct in saying that less than 6 mg/dl was the target serum uric acid after starting ULT. Moreover, 36.8% knew that anti-inflammatory prophylaxis could last from 3 to 6 months, while nearly half (46.6%) believed that anti-inflammatory prophylaxis is not mandatory. Nearly all PHPs (90.2%) knew that allopurinol was the preferred form of ULT. Most PHPs understood the importance of discussing diet and lifestyle modifications in the case of gout/hyperuricemia at the clinic (96.2%). Accordingly, the overall mean practice score was 7.00 (SD 2.04), with poor, moderate, and good practices being determined in 23.3%, 63.9%, and 12.8% of the participants, respectively.

**Table 3:** Assessment of primary care providers’ (PHPs) practice in the management of asymptomatic hyperuricemia and gout (n=133) † Variable with multiple response answers. * Indicates correct answer.

Statement	N (%)
Which of the following would you prefer to perform in the acute setting of the first gouty attack? ^†^	
Joint aspiration *	30 (22.6%)
Serum urate level	89 (66.9%)
Urate-lowering therapy (ULT)	37 (27.8%)
None of the above	20 (15.0%)
Which of the following are effective during acute management? ^†^	
Colchicine *	84 (63.2%)
Steroids *	32 (24.1%)
NSAIDs *	108 (81.2%)
None of the above	07 (05.3%)
Urate-lowering therapy (ULT) should be started upon which of the following? ^†^	
Recurrent flares *	107 (80.5%)
Asymptomatic hyperuricemia	23 (17.3%)
Tophi *	72 (54.1%)
Radiographic findings *	63 (47.4%)
The target serum uric acid after starting urate-lowering therapy (ULT) is?	
<10 mg/dl	08 (06.0%)
<8 mg/dl	27 (20.3%)
<6 mg/dl *	87 (65.4%)
<3 mg/dl	11 (08.3%)
Anti-inflammatory prophylaxis can be continued for how long?	
1 month	13 (09.8%)
<2 months	09 (06.8%)
3–6 month *	49 (36.8%)
No prophylaxis	62 (46.6%)
Which one of the following urate-lowering therapies (ULTs) are recommended over others?	
Allopurinol *	120 (90.2%)
Febuxostat	09 (06.8%)
Probenecid	03 (02.3%)
Benzbromarone	01 (0.80%)
Which of the following should be done if serum uric acid cannot be achieved by allopurinol?	
Increase the dose of allopurinol *	51 (38.3%)
Switch to febuxostat	44 (33.1%)
Add uricosuric lesinurad	18 (13.5%)
Wait	20 (15.0%)
Which of the following, regarding the modification of diet and lifestyle in case of gout/hyperuricemia, do you practice at your clinic?	
I view it as important and I discuss it with patients at the clinic *	128 (96.2%)
I view it as important but I do not have time to discuss it with patients at the clinic	02 (01.5%)
It is not part of my medical consultation	03 (02.3%)
Total practice score (mean ± SD)	7.00 ± 2.04
Level of practice	
Poor	31 (23.3%)
Moderate	85 (63.9%)
Good	17 (12.8%)

**Figure 1:**
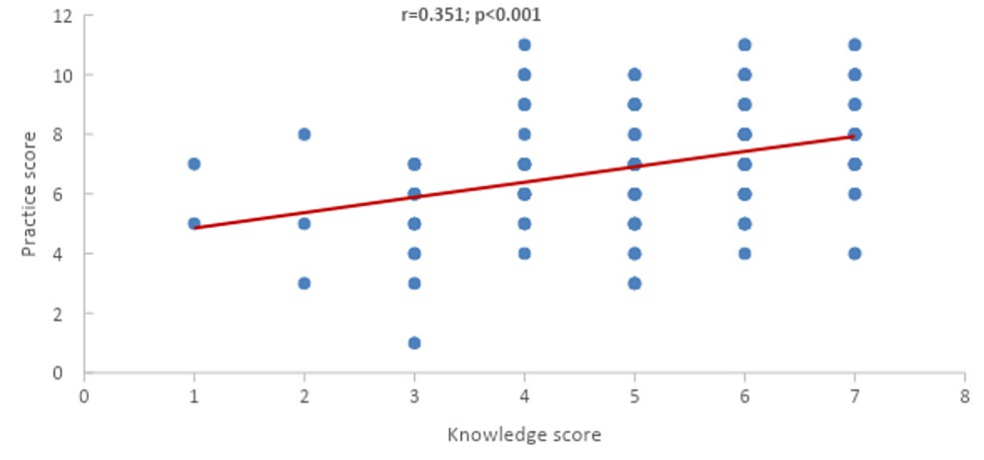
Correlation (Pearson-r) between knowledge score and practice score

Figure 1 shows an association between the practice score and the knowledge score. A significantly positive correlation between the knowledge and practice score (r=0.351; p<0.001) can be observed. Therefore, this indicates that the practice score is likely to rise whenever the knowledge score does.

When measuring the differences in the knowledge and practice scores, according to the socio-demographic characteristics of the PHPs (Table 4), it was found that a participant more likely acquired a higher knowledge score in the >45 years old group (Z=20.517; p<0.001). It is also more likely to be a participant with more than 10 years of experience (H=21.005; p<0.001) and one who attended CME on AH or gout (Z=3.594; p<0.001). Further, a higher practice score was more likely obtained by a participant in the >45 years old group (H=10.819; p=0.004) with more than 10 years of expression (H=5.740; p=0,057) and who attended CME on AH or gout (Z=2.357; p=0.018). Other variables, such as medical specialty, reading about AH or gout, and awareness of guidelines on the management of AH or gout (p>0.05), did not show significant differences in the knowledge and practice scores.

**Table 4:** Differences in the scores of the knowledge and practice according to the socio-demographic characteristics of primary healthcare providers (PHPs) (n=133) a P-value has been calculated using Kruskal-Wallis H-test. b P-value has been calculated using Mann-Whitney Z-test. ** Significant at p<0.05 level.

Factor	Knowledge		Practice	
	Score (7) Mean ± SD	Z/H-test; P-value	Score (12) Mean ± SD	Z/H-test; P-value
Age group ^a^				
25–34 years	4.43 ± 1.36	H=20.517; P<0.001 **	6.17 ± 1.84	H=10.819; P=0.004 **
35–44 years	5.15 ± 1.44		7.03 ± 2.09	
>45 years	5.92 ± 0.94		7.71 ± 1.90	
Gender ^b^				
Male	5.16 ± 1.40	Z=0.147; P=0.883	6.69 ± 2.02	Z=2.044; P=0.041 **
Female	5.21 ± 1.39		7.54 ± 1.99	
Medical specialty ^b^				
General practitioner	5.08 ± 1.49	Z=0.724; P=0.469	6.89 ± 1.97	Z=0.537; P=0.591
Family medicine	5.31 ± 1.27		7.14 ± 2.14	
Years of experience ^a^				
<5 years	4.19 ± 1.33	H=21.005; P<0.001 **	6.27 ± 1.87	H=5.740; P=0.057
5–10 years	5.06 ± 1.44		7.15 ± 2.14	
>10 years	5.70 ± 1.14		7.20 ± 1.99	
Attended CME on AH or gout ^b^				
Yes	5.84 ± 1.07	Z=3.594; P<0.001 **	7.65 ± 2.03	Z=2.357; P=0.018 **
No	4.87 ± 1.43		6.69 ± 1.98	
Read about AH or gout in the last year ^b^				
Yes	5.18 ± 1.44	Z=0.190; P=0.849	7.13 ± 1.97	Z=1.067; P=0.286
No	5.18 ± 1.29		6.69 ± 2.19	
Aware of guidelines on the management of AH or gout ^b^				
Yes	5.20 ± 1.41	Z=0.286; P=0.775	7.00 ± 2.13	Z=0.049; P=0.961
No	5.14 ± 1.39		7.00 ± 1.86	

## Discussion

This study was carried out to determine primary healthcare providers' knowledge and practice in managing AH and gout. The questionnaire questions were obtained from recent guidelines. The findings of this study suggest that although the knowledge of our PHPs is sufficient, their practice was less-than-adequate. The overall level of knowledge was good among 45.9% of PHPs. However, 40.6% possessed a moderate level of knowledge, but only 13.5% were considered to have a poor level of knowledge. The overall level was poor among 23.3%, and 63.9% possessed reasonable practice. Nevertheless, only 12.8% were considered to have a good practice. Consistent with these findings, Alharthi [9],2021, found that nearly all (86.6%) physicians possessed a good knowledge of managing AH at the primary health centers in the Bisha Province. However, their level of practice was a cause for concern, as only 43.9% had good practice, and just 27.4% of the physicians had attended CME on either gout or AH. In China [10], Liu et al., 2021, general practitioners' knowledge regarding gout were lower than our PHPs. Findings indicated that the overall rate of a good understanding of gout was only 6.5%. The basic knowledge level was 55.6%, and the understanding of gout diagnosis and treatment was only 11.1%. Data in this study revealed that the older age group (>45 years) who regularly attended CME for AH/gout exhibited significantly better knowledge and practice scores than the remaining groups. This almost concurred with the study of Liu et al. [10], 2021, whose findings suggested that general practitioners' knowledge and management of gout was higher among those who understood basic concepts related to gout. It also states that CME could improve their understanding of gout diagnosis and treatment. Further education about the causes and risks of AH and gout among healthcare providers may bridge the gaps in their knowledge and practice, ultimately leading to better management.

Moreover, increasing years of experience was associated with increased knowledge scores. However, this correlation did not extend to the practice scores, as we found no significant differences between years of experience and the level of practice. In contrast, Alharthi [9], 2021, found that years of experience and reading about AH in the last 12 months were predictors of good practice. When we compared the knowledge and practice of the medical field of each participant, we learned that the knowledge and practice of general practitioners and family physicians were not significantly different. However, we predicted that female PHPs were more likely to exhibit better practice than male PHPs, but such gender differences were not proven through literature. Thus, further investigation is needed to establish its credibility.

Interestingly, we found a significant positive correlation between the knowledge score and the practice score. Similarly, Alharthi [9], 2021, states that information based on guideline recommendations will provide better patient management plans. The prevalence of PHPs who attended CME on the subject of AH and gout was 32.3%. This figure was lower than those who read about AH or gout in the last year (70.7%) and those who were aware of the guidelines for managing AH or gout (67.7%). However, reading or awareness of such guidelines does not have a relevant effect on either knowledge or practice. This is inconsistent with the paper by Furlan et al. [7], 2021. Based on their reports, PHPs who read at least one scientific paper on hyperuricemia in the past year scored significantly higher in the knowledge test. Additionally, a study conducted by Liu et al. [10], 2021 documented that a large percentage of the general practitioners in the Tongzhou district of Beijing, China, had received CME about gout (71.8%), while more than half (54.6%) were aware of its treatment. However, despite these statistics, the author emphasized that there were severe deficits among general practitioners in understanding gout, wherein quality CME is needed to improve their management of gout.

## Conclusions

Although PHPs’ knowledge of managing AH and gout was adequate, this did not reflect in their practice. Physicians with more years of experience who attended CME on AH and gout demonstrated better knowledge and practice than the rest of the PHPs. It is necessary to address the gaps in the practice of our PHPs, which could be done through in-depth training about AH and gout. While having a good understanding of the management of hyperuricemia and gout could be an advantage, this has to be translated into real cases to decrease the risk of malpractice and the costly treatments of these conditions. Our study could guide other researchers to assess the gaps in other clinical practices that PHPs face.
